# COVID-19 related distress in the Swedish population: Validation of the Swedish version of the COVID Stress Scales (CSS)

**DOI:** 10.1371/journal.pone.0263888

**Published:** 2022-02-14

**Authors:** Anders Carlander, Mats Lekander, Gordon J. G. Asmundson, Steven Taylor, Roger Olofsson Bagge, Ann-Sophie Lindqvist Bagge

**Affiliations:** 1 SOM Institute, University of Gothenburg, Gothenburg, Sweden; 2 Wallenberg Centre for Molecular and Translational Medicine, University of Gothenburg, Gothenburg, Sweden; 3 Department of Psychology, Stress Research Institute, Stockholm University, Stockholm, Sweden; 4 Department of Clinical Neuroscience, Karolinska Institute, Stockholm, Sweden; 5 Department of Psychology, University of Regina, Regina, Canada; 6 Department of Psychiatry, University of British Columbia, Vancouver, British Columbia, Canada; 7 Department of Surgery, Sahlgrenska University Hospital, Region Västra Götaland, Gothenburg, Sweden; 8 Department of Surgery, Institute of Clinical Sciences, Sahlgrenska Academy at the University of Gothenburg, Gothenburg, Sweden; 9 Department of Psychology, University of Gothenburg, Gothenburg, Sweden; Konkuk University, REPUBLIC OF KOREA

## Abstract

**Background:**

The COVID Stress Scales (CSS) assess health- and contamination-related distress in the face of a medical outbreak like the ongoing COVID-19 pandemic. Though the CSS is translated into 21 languages, it has not been validated in a Swedish national sample.

**Aim:**

Our general objective is to provide a translation, replication, and validation of the CSS and test its convergent- and discriminant validity in relation to anxiety, health anxiety, depression, and stress in the general Swedish population. We also present latent psychometric properties by modelling based on item response theory.

**Methods:**

Participants consisted of 3044 Swedish adults (> 18 years) from a pre-stratified (gender, age, and education) sample from The Swedish Citizen Panel. Mental health status was assessed by validated instruments, including the CSS, PHQ-4, SHAI-14, and PSS-10.

**Results:**

Results indicate that our Swedish translation of CSS has good psychometric properties and consists of 5 correlated factors.

**Discussion:**

The CSS is useful either as a unidimensional or multidimensional construct using the CSS scales to measure key facets of pandemic-related stress.

**Conclusions:**

The findings support the cross-cultural validity of the CSS and its potential utility in understanding many of the emotional challenges posed by the current and future pandemics.

## Introduction

The ongoing COVID-19 pandemic has had a major toll on our lives. People are facing challenges that may cause widespread fear and stress due to the inherently uncertain nature of the pandemic crisis. It is pivotal to understand the psychological mechanisms surrounding a pandemic in terms not only of public health but also to curtail and manage the spreading of the disease. Psychological stress and anxiety have gained attention as driving emotions underlying individuals’ behavioural responses to medical outbreaks [1] since high levels of stress and anxiety affect the ability to make rational decisions [2, 3]. Taylor and colleagues [4] propose that individuals with a high level of COVID-related stress are more likely to misinterpret minor alignments as signs of serious infections, seek health care, and engage in panic buying [5, 6]. In contrast, individuals with low levels of COVID-related stress are less likely to engage in hygiene behaviors, less likely to adhere to restrictive recommendations, and are less likely to get vaccinated. Findings from studies using network analysis support this conceptualization [7, 8]. Taylor and colleagues [4] propose that the COVID-19 pandemic stimulates stress-related responses like fear of getting infected, fear of contamination through objects, fear of foreigners (i.e., disease-related xenophobia), fear of socio-economic consequences, reassuring-seeking concerning pandemic-related threats, and traumatic stress.

Taylor and colleagues [4] developed the COVID Stress Scales (CSS) to measure the facets of COVID-related stress. Development and validation strategies were undertaken in English using a large representative sample of adults for Canada and the United States. Although initially designed to comprise 36 items distributed equally across six scales assessing each of COVID-related danger (D), socio-economic consequences (SE), xenophobia (X), contamination (C), traumatic stress (TS), and compulsive checking (CH), factor analytic findings suggested a 5-factor model in which the D and C scales loaded on to a single factor (i.e., COVID-related danger and contamination fears). Taylor and colleagues [4] retained all 12 items to measure COVID-related danger and contamination fears and six items for each of the other scales. The CSS facilitates a better understanding of the distress associated with COVID-19 and the identification of people in need of mental health services. The CSS were developed during the COVID-19 pandemic but were designed to be a transdiagnostic instrument that may be modified for future pandemics or medical outbreaks [4].

The CSS has been validated against instruments assessing anxiety, depression, health anxiety, OCD, xenophobia, social desirability, and has demonstrated good reliability and validity in population-representative [4] and clinical [9] samples. The CSS has been translated to 21 languages and has become a much-used instrument in the assessment of COVID-related emotional distress. Studies reporting on the psychometric properties of the Persian, Filipino, Nepali, Arabic, Serbian, Italian, Polish, and Vietnamese CCS have been published (see https://coronaphobia.org/professional-resources/). Notwithstanding its widespread use and adaptation for various countries and cultures, the CSS have not yet been validated in a Swedish national sample. As a pandemic is a global concern, it is of utmost importance that widespread international collaborations remain a priority. This includes sharing data on COVID-19 cases, hospitalizations, and vaccine uptake. Still, it is equally important to collect data pertaining to mental health in general and specific instruments like the CSS indicating how and why people may be experiencing varying degrees of pandemic-related distress.

The present study provides a cross-cultural translation, replication, and validation of the CSS in a Swedish national sample. This may be especially important in a global comparison between countries and regions since Sweden has had a reputation for having a “relaxed” corona response relying on individual responsibility. We replicated the steps and considerations presented in the original article [4] and extended them with the added value of additional psychometric tests. Our specific aims were to:

1. Describe the translation process from English to Swedish and report any cultural considerations and deviations regarding Sweden in relation to the original Canadian and American samples.
2. Conduct confirmatory factor analysis and compare the Swedish CSS model fit to the original CSS.
3. Test convergent and discriminant validity regarding anxiety, health anxiety, depression, and perceived stress.
4. Model the underlying latent construct of CSS and explore how well each item can discriminate between high and low levels of COVID-related stress, which will allow a more in-depth diagnostic validation of this newly developed instrument.

## Method

### Participants

Data were collected through a web panel—The Swedish Citizen Panel—administered by the University of Gothenburg in Sweden (https://www.gu.se/en/som-institute). Participants consisted of a pre-stratified (gender, age, and education) population-representative sample of Swedish adult citizens (> 18 years of age) who previously had enrolled or been recruited to be a part of the web panel. An invitation to participate in the study was sent out to 5,400 individuals within the panel. The net participation rate was 57%, with 3,044 responses. There was an approximately equal number of males (51.3%) and females (48.7%). The participants were somewhat older (M = 53.8 years, SD = 16.2), had a higher educational level (bachelor degree or higher = 30.7%), and had higher income (median range = 30′-37′ SEK) compared to the general Swedish population [10]. We obtained written informed consent from the participants when entering the survey.

### Translation process

Two independent certified translators performed forward translation (English to Swedish) and back translation (Swedish to English) of the CSS. Two researchers (AC, ALB) compared the original English CSS to the back-translated Swedish version. Minor wording discrepancies were identified and resolved after re-consulting the translators. During initial pilot testing, most of the 25 selected participants from the general population expressed both in written and verbal feedback that the Swedish translation of the word ’foreigners’ (*främlingar*) created uncertainty about its exact meaning. After consultation with the translators, this translation was changed to another Swedish word for ‘foreigners’ (i.e., *utlänningar*) for items 13, 15, 17, and 18 on the X scale. For item 16 on the X scale, the Swedish translation of ‘person from a foreign country’ (*person från ett främmande* land) was changed to ‘person from another country’ (*person från ett annat land*). A second smaller pilot study of 10 participants was then conducted, and the participants reported no ambiguities about the translation. During the final data collection, the Swedish translation of ’foreigners’ (*utlänningar*) was changed to ‘people from other countries’ (*människor från andra länder*) for items 13, 15, 17, and 18, as described in greater detail below. For the final Swedish version of the CSS, see https://coronaphobia.org/professional-resources/.

### Data collection procedure

All surveys administered at the SOM Institute start with a ‘soft launch’ where an invitation to participate is sent out to a small subsample of participants to test that the data collection proceeds without problems. In the present study, after the soft launch, the data collection was paused for approximately five days when several complaints were received from participants who expressed concern that the Swedish translation of the word ‘foreigners’ (*utlänningar*) in the CSS had a racist connotation. The survey was resumed after the Swedish translation of ‘foreigners’ (*utlänningar*) was changed to ‘person from a foreign country’ and ‘people from other countries’ (see above) for items 13, 15, 17, and 18.

The data collection was carried out from February 25^th^ to March 30^th^, 2021. Two email reminders were sent after 8 and 15 days, and the entire data collection was carried out in 35 days, including the soft launch. At the time, the number of COVID-19 cases was once again on the rise in Sweden, with a total of approximately 800,000 confirmed cases and over 13,000 deaths. Still, the weekly number of fatalities had plateaued at around 130 cases (https://covid19.who.int/region/euro/country/se).

### Measures

#### COVID Stress Scales

The primary instrument of interest in this research is the CSS [4]. The CSS comprises 36 items that were initially distributed over 6 subscales [Danger (D), Socio-economic consequences (SE), Xenophobia (X), Contamination I, Traumatic stress (T), and Compulsive checking (CH)] that assess different facets of COVID-19-related distress over the past seven days. Based on Taylor et al. [4], the subscales of D and C loaded on the same factor which renders that the final CSS consist of the five subscales D, SE, X, TS, and CH. The D-scale consists of 12 items, whereas the other scales (SE, X, TS, and CH) consist of six items, all rated on a 5-point scale. The scales are distinct but highly correlated [7]; therefore, both total and scale scores can be computed, with higher scores indicating greater COVID-19-related distress. Current evidence supports the validity and reliability of the CSS (e.g., [4, 9]).

#### Validation instruments

Instruments and single-item questions assessing the level of self-reported anxiety, health anxiety, depression, and stress were used to assess convergent and discriminant validity. These measures were generic and not tied to COVID-19. We use two of the instruments that Taylor et al. [4] used when validating the CSS, including the Patient Health Questionnaire-4 (PHQ-4); [11] and the Short Health Anxiety Inventory (SHAI-14); [12]. To further validate the CSS, we added the Perceived Stress Scale (PSS-10) [13].

The PHQ-4 was developed as a screening instrument for anxiety and depression [11] and has been demonstrated to be a reliable and robust instrument with normative data for general populations [14]. The PHQ-4 consists of two statements measuring anxiety (e.g., “Feeling nervous, anxious or on edge”) and two statements assessing depression (e.g., “Feeling down, depressed, or hopeless”), all scored using a 4-point rating scale ranging from 0 (Not at all) to 3 (Nearly every day). The PHQ-4 has demonstrated good reliability and validity in clinical and non-clinical samples [11, 14].

The 64 item Health Anxiety Inventory and the shorter 14-item SHAI employed in this study were developed to be sensitive to general health concerns and more pathological health anxiety [12]. The SHAI-14 comprises 14 questions, each consisting of four statements that participants are asked to choose from based on level of agreement (e.g., “I do not worry about my health”). The SHAI-14 has demonstrated good reliability and validity across non-clinical and clinical samples [12, 15, 16].

Stress was assessed by the Perceived Stress Scale, which was originally published as a 14-item instrument (PSS-14) [17]; but, a shorter 10-item version (PSS-10) was later introduced, showing better psychometric properties [13]. The PSS-10 measures the extent to which persons appraise that their demands exceed their ability to cope during the last month. The PSS-10 consists of ten positively or negatively phrased questions (e.g., “In the last month, how often have you been upset because of something that happened unexpectedly?”), which are scored using a 5-point rating scale ranging from 0 (Never) to 4 (Very often). PSS-10 has been validated across different languages and cultures with good psychometric properties [18], including Swedish samples [19].

#### Demographics and socio-economic status

Gender was coded as male (0) and female (1). Age corresponds to participants’ reported age, ranging from 18–86 years. Level of educational attainment was assessed using a 9-point rating scale ranging from 1–2 = elementary, 3 = high school <3 years; 4 = high school>4 years, 5–6 = post-secondary non-college/vocational, 7 = college<3 years, 8 = college>3 years, and 9 = graduate school/PhD. Personal monthly income was assessed on a 13-point rating scale ranging from 1 = less than 4,000 SEK to 13 = more than 65,000 SEK.

### Ethical approval

The study has ethical approval by the regional Swedish Ethical Review Authority (D-nr 948–18). All participants signed informed consent before entering the study.

### Statistical considerations

Since this is a replication and validation study, we followed the steps and included the related constructs in relation to the original manuscript published by Taylor et al. [4]. We conducted a confirmatory factor analysis (CFA) to show the goodness of fit of both the original theoretical six-factor model and the empirical five-factor model from the EFA presented by Taylor et al. [4]. In accordance with Taylor et al. [4], we assessed convergent and discriminant validity by evaluating correlations between CSS, PHQ-4, and SHAI-14. Moreover, we tested the correlation between the CSS and PSS-10. We reported a multiple ordinary least squares (OLS) regression analysis showing the CSS regressed on sex, age, education, and income to determine how demographics and socioeconomic status may be related to CSS scores. Finally, we analysed the 36 CSS items using item response theory (IRT) to evaluate how each item discriminates between high and low stress levels on a latent construct of the CSS.

## Results

### Descriptive results

Since the data collection was halted after the initial soft launch and the wording was altered on questions with connotations to xenophobia in contemporary Swedish (described above), we conducted pairwise comparisons between the soft launch (n = 158) and the rest of the sample (n = 2869), finding that X had significantly lower values (t(2871) = 3.62, p = < .001) in the soft launch sample (M = 2.75, SD = 4.57) compared to the rest of the sample (M = 4.30, SD = 5.17). Accordingly, we decided to omit the soft launch data from the rest of the analyses. The final sample size was, therefore 2869 participants. Our sample presented with anxiety (M = 1.05, SD = 1.48) and depression (M = 1.26, SD = 1.56) scores that were well below the cut-off threshold of ≥3 in PHQ-4 [11]. For health anxiety, there are some uncertainties regarding cut-off scores [15]; nonetheless, the relatively low score of our sample generally implies an absence of pathological health anxiety (M = 9.75, SD = 5.95).

Following Taylor et al. [4], we assessed the 5-factor model that was rendered from their exploratory factor analysis (EFA) as well as the original 6-factor model. We present mean (crude mean score), dispersion (standard deviation), normality (skewness and kurtosis), and internal consistency (McDonald’s ω) for the total CSS and each scale (Table 1). As can be seen, there are some deviations from normality in the scales compared to an ideal normal distribution of skewness = 0 and kurtosis = 3/0 [20]. In terms of internal consistencies of each scale, we report McDonald’s ω, which is similar but regarded superior to the more common alpha, and ω >.8 indicates a good reliability [21]. The CSS generally have good reliability in our data, albeit CH has the lowest estimate.

**Table 1:** Descriptive statistics of CSS (total and the six subscales Danger (D), Socio-economic consequences (SE), Xenophobia (X), Contamination (C), Traumatic stress (T), and Compulsive checking (CH): Mean score, standard deviation, normality (skewness and kurtosis), internal consistency (McDonald’s ω) and correlation.

Scale	M	SD	Sk.	Ku.	ω	Pearson correlation^a^					
**CSS**_**total**_	23.12	17.53	1.33	5.30	.94	**1**	**2**	**3**	**4**	**5**	**6**
**1. D**	7.38	5.24	.62	2.77	.88						
**2. SE**	1.44	2.89	3.22	15.95	.89	.42					
**3. X**	4.30	5.17	1.56	5.00	.92	.50	.39				
**4. C**	5.64	4.62	1.11	4.14	.89	.66	.35	.59			
**5. T**	1.96	3.10	2.41	9.63	.88	.50	.40	.32	.49		
**6. CH**	2.39	2.81	1.70	6.52	.72	.36	.32	.23	.33	.47	

^a^All correlations significant at p < .001

### Confirmatory factor analysis

We tested the model fit of both the original 6-factor model and the 5-factor model from Taylor et al. [4] that was indicated from their exploratory factor analysis (EFA). Both models performed well; but the 6-factor model indicated a slightly better goodness-of-fit: RMSEA = 0.069 (90% CI: 0.067–0.070), SRMR = 0.054, and CFI = .88 compared to the 5-factor model: RMSEA = 0.078 (90% CI: 0.076–0.079), SRMR = 0.059, and CFI = .84. No alternative models or parameter constraints were included, but an additional postestimation test indicated that several modification indices (MI) exceeded the rule-of-thumb-based value of 100. Most notably, in the present sample, the latent variable of D and items 21, 22, and 23 indicated deviations from the specified 6-factor model, both in terms of the measurement model and correlated error terms—specifically, items 21, 22, and 23 indicated loadings from most latent scale constructs. The covariance structure indicated a large portion of variance in these three items that remain unaccounted for by the latent construct and the scale of D alone. This may indicate that D entails yet another unspecified subscale or a hierarchical order in which a superordinate construct exerts influence over the D scale.

### Convergent and discriminant validity

Following Taylor et al. [4], the results show that the correlations between CSS, health anxiety, depression, anxiety, and perceived stress vary between small to medium. The TS scale generally displays the strongest associations among the CSS subscales (Table 2).

**Table 2:** Pearson correlations of the total score of CSS and the six subscales (D, SE, X, C, T, CH) and health anxiety (SHAI-14), anxiety (PHQ-4), depression (PHQ-4) and perceived stress (PSS-10).

Instrument	Covid Stress Scales^a^						
	CSS_total_	D	SE	X	C	T	CH
**SHAI-14**	.48	.41	.27	.24	.39	.51	.33
**PHQ-4 (anxiety)**	.31	.22	.26	.09	.24	.46	.23
**PHQ-4 (depression)**	.31	.24	.24	.13	.22	.44	.23
**PSS-10**	.35	.27	.27	.14	.26	.45	.25

^a^All correlations significant at p < .001

### Demographic and socioeconomic predictors of CSS

We conducted a multiple OLS regression analysis to investigate how sex, age, education, and income related to the CSS. As shown in Table 3, sex is not significantly associated with the CSS; but higher age demonstrates a small albeit significant effect in that higher age is related to higher CSS. Furthermore, higher education and income are inversely associated with CSS. However, only approximately 3 percent of the variance in CSS can be explained by the predictors in our regression model.

**Table 3:** Multiple ordinary least squares (OLS) regression analysis showing the Covid Stress Scales (CSS) regressed on sex, age, education, and income.

	*β*	*B (SE)*	*p*	*95% CI*	
				Lower	Upper
**Man**	Base				
**Woman**	.02	.81 (.74)	.274	-.64	2.26
**Age**	.06	.07 (.02)	.004	.02	.11
**Education**	-.06	-.54 (.19)	.005	-.93	-.16
**Income**	-.12	-.68 (.13)	.000	-.93	-.44
**Constant**		27.64 (2.04)	.000	23.64	31.65

Note: Adjusted R^2^ = .03; F(4, 2271) = 15.96, p < .001

### Item response theory

We conducted an additional Item Response Theory (IRT) analysis [22]. The purpose was to model the underlying latent construct theta, (*θ*) of CSS. However, since the CSS comprise several underlying subscales, we cannot formally ascertain that the general IRT assumption of uni-dimensionality holds nor that the items are locally independent (due to correlated residuals in the CFA). We fit a graded response model (GRM) since the rating scales used in the CSS may be considered ordered polytomous categories, ranging from low too high in level of agreement. The results indicate that each CSS-item can discriminate well between low and high levels of the latent construct of *θ*, the discrimination parameter *a* varies between .56 and 2.69 (see S1 Table for full table).

**Fig 1:**
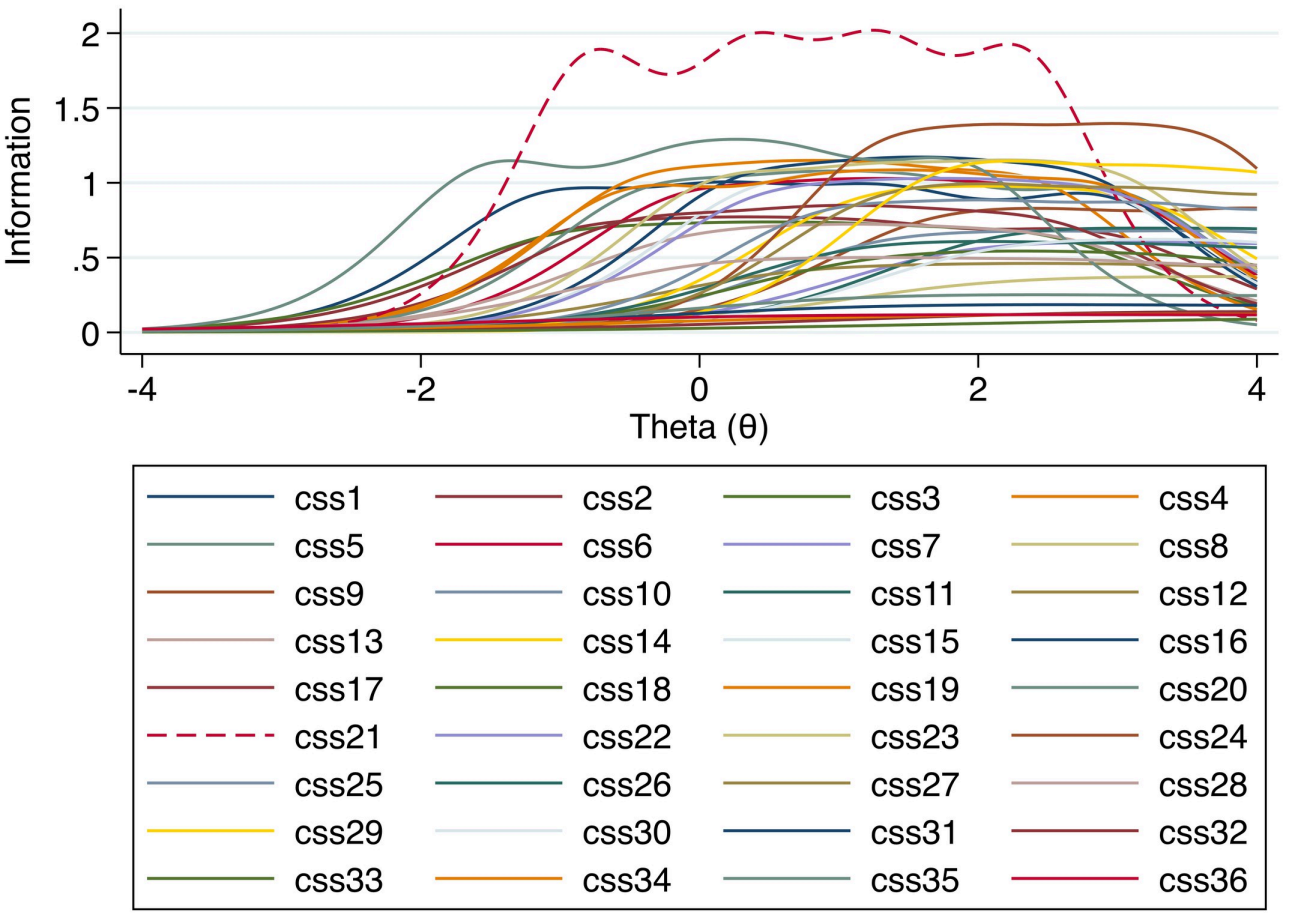
Item Information Function (IIF) of CSS. The Item Information Function (IIF) indicates how much information each CSS-question (css1-css36) can provide from the latent modelled construct of Theta (in this case corresponding to a general COVID-related stress). Some specific questions may provide more information at a relatively higher, or lower, level of Theta depending on for example question specificity and phrasing. The level Theta is standardized and in this particular case levels higher than zero on the x-axis may be assumed to assess a higher degree of COVID-related stress compared to values lower than zero where the COVID-related stress may be less salient. The y-axis represents level of information and indicates where on the Theta scale each item is most sensitive. In Fig 1 we can see that most items have a curve that is flat at the top which entails that they can provide the same amount of information at a relatively wide range of Theta. For example, item 21 provides the most information compared to the other items, and it demonstrates approximately the same level of sensitivity from a level of nearly -2 to 2 of Theta.

The level of item precision in estimating the *θ* of CSS is referred to as information within the IRT framework. If the amount of information is plotted against *θ*, an Item Information Function (IIF) is rendered. A visual inspection of the IIF in Fig 1 shows that the 36 CSS items estimate the *θ* of CSS at different locations. Still, the items are generally more reliable towards the higher end of the spectrum. This may imply that the CSS have more precision for individuals expressing a higher degree of COVID-related stress. Notably, item 21 (dashed in the figure) seems to correspond to relatively more information over a broader range of *θ*.

**Fig 2:**
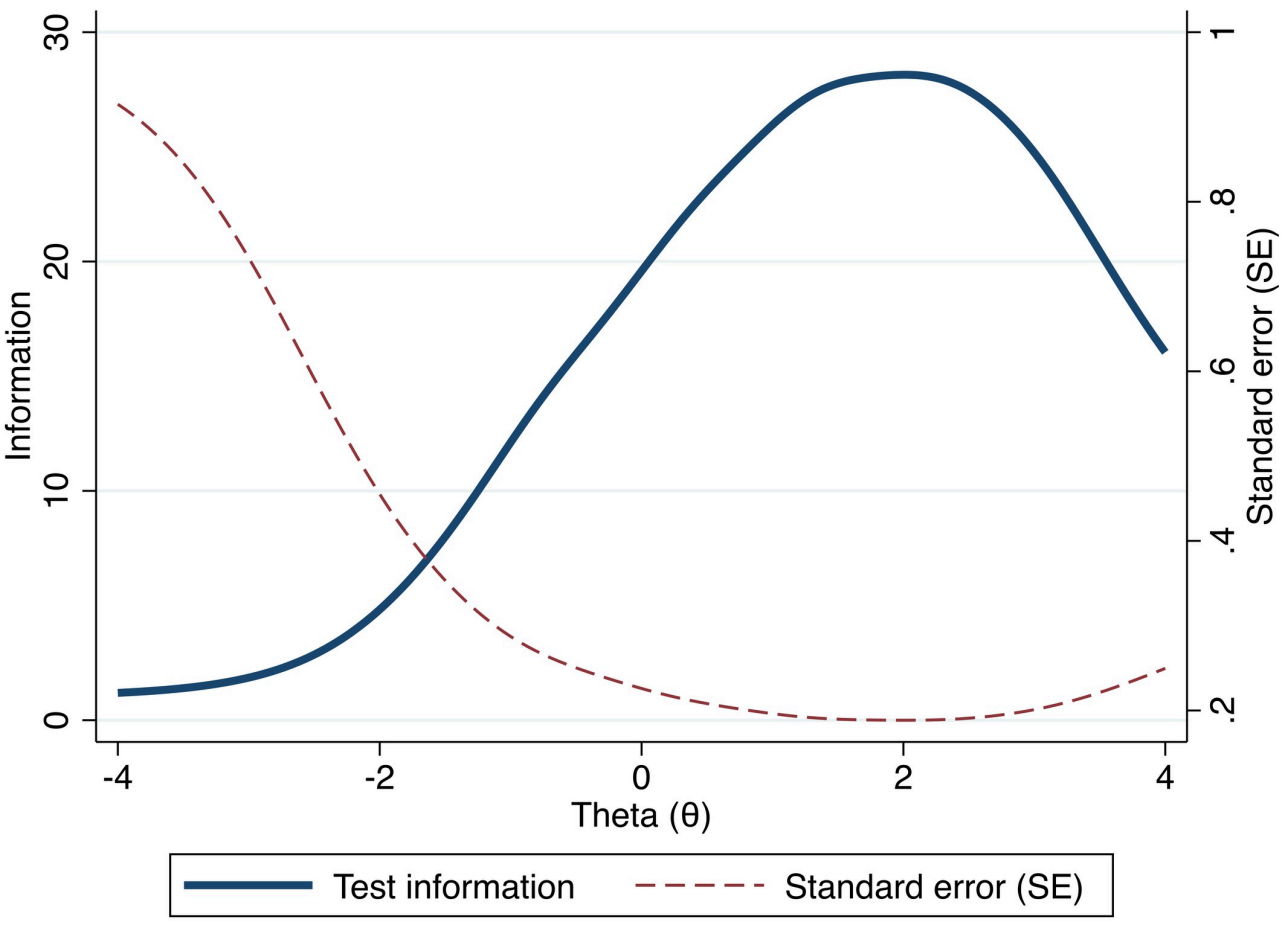
Test Information Function (TIF) of CSS. The Test Information Function (TIF) is an aggregate measure of the Item Information Function (IIF) presented in Fig 1. The TIF shows how the entire instrument (css1-css36 combined) performs at different levels of Theta and at what level or magnitude of the latent construct that we can retrieve as much information as possible. Fig 2 shows that the CSS instrument provides the most information approximately at a Theta level of 2 and, conversely, that the margin of error (SE) is at the lowest point at this particular level, meaning that the precision of the CSS-questions is likely higher at a higher level of COVID-related stress.

In relation to the IIF testing each item, the level of information extracted from the entire CSS set of items is referred to as a Test Information Function (TIF) and can be considered an aggregate model of the IIF. This means that more items equal more information of the latent construct and, in general, higher reliability. As can be seen in Fig 2, the CSS is the most reliable at *θ* = 2. At lower levels of *θ*, the Standard Error (SE) increases rapidly, indicating that the CSS may be less reliable at measuring lower levels of COVID-related stress.

## Discussion

Following the development and validation of the CSS by Taylor et al. [4], our aim was to translate and validate the CSS in a population-representative sample from Sweden. We replicated the general results reported by Taylor et al. [4], with minor deviations. Most notably, our results support that the CSS can be characterized by a stable 5-factor model in line with the recommendations of Taylor et al. [4] to merge D and C into one scale assessing danger and contamination fears. The subscales are highly intercorrelated and most items indicate a consistent degree of discrimination, and seem to be more reliable (i.e., lower error margin) at higher levels of the latent construct of CSS. There were however some deviations in the measurement model and correlated error terms, most notably pertaining to the D subscale, which indicated that the items were not fully unaccounted for by the latent construct of D alone. This may be indicative of a higher-order construct, such as a COVID-19 Stress Syndrome, which Taylor et al. [4] also suggest and have confirmed in subsequent investigations [7, 8]. A practical implication is that the CSS may be analysed either using its scale scores or as a unitary CSS total score, depending on situations and suitability. Our general results indicate that the CSS is an instrument with good psychometric properties that may offer a transdiagnostic tool for the assessment of health-related emotional distress in relation to contagions, outbreaks, and pandemics in the future.

Another aim of the present study was to test convergent and discriminant validity of CSS in relation to anxiety, health anxiety, depression, and perceived stress. When comparing our results with Taylor et al. [4] we see that the intercorrelations between scales are notably higher across the board in their Canadian and American samples compared to our Swedish sample. Concerning anxiety and depression (as measured by PHQ-4), Taylor et al. [4] report numerically stronger correlations between CSS and anxiety and depression than what is evident in our results. It is, of course, hard to compare effect sizes between studies; but we can establish that while there is an association between CSS, anxiety, and depression in the present sample, they do not seem to measure the same underlying latent constructs. For the convergent validity between the CSS scales and the measure of health anxiety (measured by SHAI-14), we observe that the correlations are relatively similar when compared to Taylor et al. [4] in terms of effect sizes. It should be acknowledged that we did not include a pre-measure of health anxiety like Taylor et al. [4]. We added yet another measure of convergent validity to compare how the CSS may be associated with perceived stress (PSS-10) and observed small to medium sized correlations. The T scale of the CSS demonstrates the strongest correlation with the PSS-10, and we conclude that while both instruments assess distress, the CSS may be more inclined towards health-related stress as opposed to stress in general.

Taylor et al. [4] showed that age and income level were negatively correlated with the total CSS score in samples from the United States and Canada, where higher CSS total scores were observed in women and in people who were unemployed and who were less educated (i.e., did not have a college education) as compared to men and people with employment and higher education. In the present sample, a limited effect of sex, age, education, and income on the variation in the Swedish version of CSS was indicated. However, higher age was related to higher CSS, and higher education and income were inversely related to CSS, in line with findings of Taylor et al. [7]. These results may imply that lower age and higher socioeconomic status may act as protective factors in the face of COVID-related stress.

An incidental finding pertaining to the translation and cross-cultural sensitivity regarding xenophobia was evident in our sample. Participants in the soft launch strongly opposed the usage of the word ‘foreigners’. As Taylor [1] has pointed out, xenophobia is a natural element of a pandemic. Here, such ingrained tendencies might fuel xenophobic tendencies towards countries or a hypothetical “patient zero”. There is evidence showing that the COVID-19 pandemic has elicited negative anti-Asian attitudes that might not necessarily be driven by racism, but by distress and concerns about the virus [23]. It may, therefore, be considered a pedagogical challenge for researchers to present a scale measuring xenophobia without causing offense.

The CSS was constructed based on knowledge and clinical experience on earlier pandemics and outbreaks [1, 4]. As such, the utility of the CSS encompasses the present COVID-19 pandemic, across all known and future variants of SARS-Cov2, as well as future outbreaks to a high degree. The CSS provides an important insight into the determinants of people’s pandemic-related emotional responses and may thus aid governmental officials in constructing suitable strategies and how to communicate to the public. In a post-pandemic world, there is a risk of significant mental health needs emerging in the public and the CSS may be used as a prognostic tool for public health officials in allocating resources, but it may also be used in clinical practice when designing more patient-specific mental health interventions.

Beyond the caveats already highlighted by Taylor et al. [4], most importantly that the development of CSS did not include structured diagnostic interviews, this study employed an online panel for the data collection which is often considered less reliable compared to a probability sample invited by postal surveys. However, the Citizen Panel is a high-quality panel, managed by a research institute within the University of Gothenburg and should be considered adequately representative of the Swedish population. In addition, contrary to other online panels, the Citizen Panel may be skewed towards older and socially and economically advantaged and more established participants. A second limitation is that the translation of an instrument may contain some cultural biases which can affect the results, due to factors like national differences in technological adaptation, demographic composition, and health insurance. Third, as mentioned above, the CSS was developed and the data were collected in a global situation where levels of anxiety, distress, and awareness likely was elevated and salient. It is, therefore, important to re-assess the psychometric properties of CSS under non-pandemic circumstances as well, improving further the possibility to gauge health and contamination distress during future infectious global challenges.

## Conclusion

The current analyses support the validity of a Swedish translation of the CSS in a population representative sample. The findings also support the cross-cultural validity of the CSS and its potential utility in understanding many of the emotional challenges posed by the current and future pandemics.

## Supporting information

S1 Table Full table of the Graded Response Model (GRM) of the Covid Stress Scales. The table shows item discrimination parameter (a), Standard Error (SE), z, 95% confidence interval (CI) and the log likelihood estimate (LL).
(PDF)
