# Supplementary material for: COVID-19 related distress in the Swedish population: Validation of the Swedish version of the COVID Stress Scales (CSS)
Source: PLoS One. 2022 Feb 14;17(2):e0263888. doi: 10.1371/journal.pone.0263888 (PMC8843112; doi:10.1371/journal.pone.0263888)
Supplement: S1 Table — The table shows item discrimination parameter (a), Standard Error (SE), z, 95% confidence interval (CI) and the log likelihood estimate (LL). (PDF) [file pone.0263888.s001.pdf]

	a	*SE*	*z*	95% CI	
css1	1.87	0.06	30.20	1.75	1.99
css2	1.59	0.06	28.52	1.48	1.70
css3	1.55	0.05	28.58	1.45	1.66
css4	1.94	0.07	29.79	1.81	2.07
css5	1.89	0.07	29.10	1.77	2.02
css6	1.84	0.07	28.32	1.72	1.97
css7	1.39	0.08	17.27	1.23	1.55
css8	1.09	0.07	15.24	0.95	1.23
css9	1.65	0.09	18.47	1.48	1.83
css10	1.49	0.07	19.95	1.34	1.63
css11	1.50	0.09	15.77	1.31	1.68
css12	1.21	0.06	21.31	1.10	1.33
css13	1.51	0.06	25.90	1.40	1.63
css14	1.76	0.08	21.98	1.60	1.92
css15	1.86	0.07	25.78	1.72	2.01
css16	1.94	0.07	26.45	1.80	2.09
css17	1.65	0.06	27.94	1.54	1.77
css18	1.31	0.07	19.70	1.18	1.44
css19	1.91	0.07	29.21	1.78	2.03
css20	2.08	0.07	31.21	1.95	2.21
css21	2.69	0.09	31.42	2.52	2.86
css22	1.82	0.07	25.79	1.68	1.96
css23	1.95	0.07	27.33	1.81	2.09
css24	2.15	0.10	21.17	1.95	2.35
css25	1.73	0.08	23.05	1.58	1.88
css26	1.41	0.07	21.15	1.28	1.54
css27	1.81	0.09	20.85	1.64	1.98
css28	1.29	0.05	23.85	1.18	1.39
css29	1.93	0.10	18.41	1.73	2.14
css30	1.42	0.09	15.78	1.24	1.59
css31	0.78	0.05	15.55	0.68	0.88
css32	0.68	0.06	11.29	0.56	0.80
css33	0.56	0.07	8.40	0.43	0.69
css34	0.63	0.05	12.89	0.53	0.72
css35	0.91	0.05	17.14	0.81	1.01

LL = -81075.39. For z, all $p<.001$.
